# Quality of life improved following in-patient substance use disorder treatment

**DOI:** 10.1186/s12955-015-0231-7

**Published:** 2015-03-14

**Authors:** Adrian R Pasareanu, Anne Opsal, John-Kåre Vederhus, Øistein Kristensen, Thomas Clausen

**Affiliations:** Addiction Unit, Sørlandet Hospital HF, PO Box 416, 4604 Kristiansand, Norway; University of Agder, Kristiansand, Norway; Norwegian Center for Addiction Research, University of Oslo, Oslo, Norway

**Keywords:** Quality of life, Substance use disorder treatment, Compulsory hospitalization, Treatment outcomes

## Abstract

**Background:**

Quality of life (QoL) is increasingly recognized as central to the broad construct of recovery in patients with substance use disorders (SUD). However, few longitudinal studies have evaluated changes in QoL after SUD treatment and included patients with SUD that were compulsorily hospitalized. This study aimed to describe QoL among in-patients admitted either voluntarily or compulsorily to hospitalization and to examine patterns and predictors of QoL at admission and at 6 months post treatment.

**Methods:**

This prospective study followed 202 hospitalized patients with SUD that were admitted voluntarily (N=137) or compulsorily (N=65). A generic QoL questionnaire (QoL-5) was used to assess QoL domains. Regression analysis was conducted to identify associations with QoL at baseline and to examine predictors of change in QoL at a 6-month follow-up.

**Results:**

The majority of patients had seriously impaired QoL. Low QoL at baseline was associated with a high psychiatric symptom burden. Fifty-eight percent of patients experienced a positive QoL change at follow-up. Although the improvement in QoL was significant, it was considered modest (a mean 0.06 improvement in QoL-5 scores at follow-up; 95% confidence interval: 0.03 - 0.09; p<0.001). Patients admitted voluntarily and compulsorily showed QoL improvements of similar magnitude. Female gender was associated with a large, clinically relevant improvement in QoL at follow-up.

**Conclusions:**

In-patient SUD treatment improved QoL at six month follow-up. These findings showed that QoL measurements were useful for providing evidence of therapeutic benefit in the SUD field.

## Introduction

The concept of quality of life (QoL) is used in medicine for measuring a patient’s subjective view of overall well-being. It serves as a complementary perspective to a traditional disease-specific perspective. The most promising use of the QoL concept is as an outcome measure in clinical trials [1] and health services research [2]. This broad evaluation is particularly useful in the context of chronic disorders, where it is often possible to improve patient living conditions, though a complete absence of symptoms may be out of reach [3]. With the recognition that measures of disease status alone are insufficient to describe the burden of illness in chronic and severe disorders, clinical research has rapidly employed QoL as an integral outcome variable [4].

Substance use disorder (SUD) is often considered a chronic, relapsing disease that is typically associated with psychiatric, somatic, and social comorbidities, in addition to a shortened life expectancy [5]. Traditionally, addiction treatment has focused on abstinence from substances; however, this “narrow” aim for treatment efficiency has recently been criticized. Increasingly, the addiction field is recognizing the importance of focusing on other positive treatment outcomes and recovery [6,7]. Recovery has been generally considered a period of time characterized by an enduring reduction in substance use, improved personal health, and improved social function. Thus, QoL is also relevant to SUD, because it is a construct that incorporates the individual’s subjective view of a range of clinical, functional, and personal variables [8].

Although SUD is difficult to cure, effective treatments are currently available to stabilize patients and reduce harm, thereby increasing life expectancy and QoL [9].Chronic illnesses have been treated for indeterminate periods, and treatment effects are typically evaluated during the course of those treatments [7]. Monitoring the outcome with specific measures of recovery can produce more efficient, clinically relevant, accountable evaluations. Applying this methodology to the SUD field has given rise to expectations of similar accuracy in evaluating outcomes. Moreover, it has been suggested that these outcomes should be collected and reported immediately and regularly by clinicians from the beginning of addiction treatment sessions, to support evaluations of recovery progress and decision-making with regard to continuing care [7]. Currently, there is evidence that QoL will improve as a function of treatment and recovery in patients with SUD [10-12]. Emerging changes in the SUD treatment field will require the incorporation of QoL indices in treatment development and research [13].

Despite relatively little research that focuses on QoL among the SUD population, it has been shown that QoL is consistently low among individuals with SUD that actively seek treatment compared to individuals without SUD or those with chronic psychiatric conditions [14]. However, few longitudinal studies exist; thus, it remains unknown whether this trend will continue to be positive through a follow-up stage. This question requires following patients for extended periods of time [15]. In Norway, it is also particularly important to evaluate outcomes for patients that were compulsorily admitted to a hospital. Despite 20 years of practice under a “Compulsory Treatment Act”, little is known about the outcomes of these patients. The Norwegian Municipal Health Care Act, § 10.2 (NMHCA) sanctions involuntary interventions for non-psychotic adult patients with SUDs [16]. The Act covers an option for retention (up to three months), when the health of the patient is seriously at risk due to extensive, prolonged substance use and voluntary efforts have proven insufficient. The formal decision for compulsory hospitalization is made by the County Committees, a local board of social welfare, consisting of legal experts and laypersons. The specialist health service must take care of these patients in increasing numbers, although both the criteria for compulsory hospitalization and for what further treatment should be offered are ill defined in the law-texts [17].

### Aims

The aim of this study was to describe QoL in a cohort of inpatients admitted to voluntary or compulsory hospitalization for SUD, typically with comorbid psychiatric disorders. Additionally, we aimed to examine changes in QoL at 6 months post treatment, and identify predictors of those changes.

## Methods

### Setting and procedures

This prospective study followed patients with SUD that were voluntarily and compulsorily hospitalized. The patients were recruited from three different publicly funded treatment centers in the southeastern part of Norway. The centers were located in Kristiansand, Tønsberg, and Oslo. The treatment wards had multidisciplinary staffs, including psychiatrists, psychologists, social workers, occupational therapists, specialized nurses, and other trained staff. The centers offered treatment for patients with primary SUD, often combined with mental disorders (except psychosis). The patient population was drawn mainly from urban and suburban areas.

In Norway, patients with SUD that are compulsorily admitted (CA) and voluntarily admitted (VA) to care are often treated in a single, gender-mixed ward. In the acute phase, the main target for the retention of the CA patients is to provide life-saving treatment; over the long term, the aim is to motivate them to enter voluntary treatment [18]. Treatment included assessments of somatic and mental health. Treatment also included pharmacotherapy; cognitive milieu therapy; and individual motivation enhancement, rather than isolating the patients.

Recruitment for the study continued consecutively from January 1, 2009 to May 31, 2011. The criteria for inclusion were as follows: substance use disorder, age ≥ 18 years, understanding/speaking the Norwegian language, and admitted at least 3 weeks prior to study inclusion allowing them enough time for stabilization. Before study inclusion, the patients were either detoxified, which was verified by negative urine tests for alcohol, opioids, central stimulants (amphetamines, methamphetamines, and cocaine), benzodiazepines, and cannabis; or they spent a minimum of 14 days in detoxification to establish baseline values not influenced by withdrawal symptoms. Patients with cognitive disabilities were excluded when they could not understand the questionnaires. Because pregnant patients with SUD were treated in special wards, they were not included in this study. Follow-up interviews were performed 6 months after discharge from the hospital, and took place in July 2009 through December 2011.

### Participants

A total of 326 patients were identified as potentially relevant for this study, but only 228 were eligible, due to various reasons, including insufficient mental capacity, a short stay, or logistical issues. Twenty six refused to participate. Thus, 202 patients were enrolled in the study. Among these, 65 were CA and 137 were VA. The follow-up was conducted at 6 months after discharge from treatment and 123 patients were reached at follow-up (61%). Significantly more CA patients were included at follow-up (82% versus 59%). This was due to financial constraints in the study and the large geographical uptake area. As patients came from all over the country, it was deemed necessary to prioritize to reach the CA patients nationwide, as compulsory admission was a variable of particular interest for this study. Thus, the higher loss to follow-up in the VA group had administrative and logistic reasons. An attrition analysis showed that there were no differences between those who dropped out and those who were reached at follow-up on demographic data, severity scores or length of stay.

### Instruments and measures

The Mini International Neuropsychiatric Interview (MINI), version 5.0, was conducted at baseline to confirm the SUD diagnosis [19]. In the analysis, SUD diagnosis was dichotomized to alcohol use disorder or drug use disorder. Those with both alcohol and drug use disorder were coded as alcohol use disorder. Demographics were recorded. Substance use variables were assessed based on the European Addiction Severity Index (EuropASI), a structured interview designed for both clinical and research purposes [20].

Psychiatric symptom burdens were measured with the Symptom Checklist-90-R (SCL-90-R), which contains 90 items, and measures 9 primary symptom dimensions that provides an overview of a patient’s symptoms and their intensity. Each of the 90 items is rated on a five-point Likert-type scale, ranging from “not at all” (0) to “extremely” (4): higher values indicate greater symptom severity during the past week. The Global Symptom Index (GSI) score in SCL-90-R was used to assess the level of general psychological distress [21]. A cut-off score of GSI>1 was used as a general measure of psychopathology [22].

Quality of life was measured with the QoL-5, a generic QoL instrument intended to measure satisfaction with life in general; i.e., it is not disease-specific. Generic instruments are preferred in diseases with multidimensional consequences, like SUD [23]. QoL-5 is based on the integrative theory of the QoL concept [24] and consists of five subjective QoL statements; two questions about health, physical and mental; two questions about the quality of the relationship with important others (partner and friends); and one question about existential QoL, i.e., the relationship with oneself. Responses were based on 5-step ordinal scales that varied from 1=very good to 5=very bad. The raw scores were transposed into a decimal scale, where 1 = 0.9 (the highest/best score) and 5 = 0.1 (the lowest/worse score) [25]. Mean scores for health, relationships, and existential QoL aspects were calculated, and the total QoL score was calculated as the mean of these three scores. When the patient did not have a partner, the relationship subscore was calculated based on one question. Normative data from a general population sample showed a mean QoL score of 0.69 [26]. The cut-off score for a markedly low QoL was suggested to be a score below 0.55, and an extremely low QoL score was < 0.4 [27]. Changes in QoL were computed by subtracting the QoL determined at admission from the QoL determined at follow up, hereafter called the ‘QoL-5 score change’. Thus, a ‘positive score change’ refers to an improved QoL. A 0.2 or higher score improvement was considered to be a large, clinically important improvement; other improvements were considered moderate (≥0.1 score), small (≥0.05 score), or very small (<0.05) [23,28].

#### Treatment variables

Two treatment variables were evaluated in the analyses: the number of days in treatment and the type of admission to the hospital: voluntary or compulsory.

#### Follow up variables

The same variables that were used at baseline were measured again at the 6-month follow up. Additionally, the EuropASI was used to measure patient substance use patterns or abstinence and the number of days spent in a controlled environment/treatment during the 30 days preceding the interview.

#### Missing data

Seven of the 202 participants did not provide QoL-scores at intake (Table 1). Of the 123 patients reached at follow-up, one did not proved QoL data. However, due to missing QoL-scores at intake, only 118 had QoL-scores at both intake and follow-up, which the longitudinal results are based on.

**Table 1:** **Baseline socio-demographic variables, quality of life, and mental stress scores for patients with substance use disorder**

**Variables**	**Number of patients, N (%) or** ***mean (SD)***
Total number of patients, N	202
Mean age, years	*30.0 (8.9)*
Female gender	68 (34)
Education, years	*10.8 (1.9)*
Relationship status, single (N=198)	136 (69)
Main diagnosis	
Alcohol use disorder	16 (8)
Both alcohol & drug use disorder	18 (9)
Drug use disorder	168 (83)
Severity scores	
Injection use^a^ (N=195)	105 (54)
Duration of most problematic substance use, years	*11.1 (7.6)*
Global Score Index (SCL-90R – GSI)^b^	*1.2 (0.69)*
Treatment variables	
Time in treatment, days	*57 (26)*
Compulsorily admitted	65 (32)
Quality of life (QoL-5 score) (N=195	*0.50 (0.16)*

^a^For the 6 months prior to admission.

^b^SCL-90-R – GSI: Symptom Check List-90-revised, Global Symptom Index.

### Ethics

The study was approved by The Regional Committee for Research Ethics in Norway (REK 08/206d, 2008/2900, 09/2413) and by the Privacy Issues Unit, Norwegian Social Science Data Services (NSD no. 18782). Written informed consent was obtained from all study participants.

### Analysis and statistical methods

Continuous variables are reported as means and standard deviations (SD). Categorical variables are reported as frequencies. Linear regression was performed to explore factors that were associated with QoL at baseline. Results are presented as β-values with 95% confidence intervals (95% CI). To examine predictors of QoL score changes, logistic regression was performed. The QoL-5 score change was dichotomized into groups of high and low score changes, with a cutoff value of ≥ 0.2 (i.e., a large and clinically relevant QoL change). From bivariate analysis, variables with a p-value < 0.2 were included in the multivariate analysis [29]. Results are presented as the odds ratio (OR) with 95% CI. P-values <0.05 were considered statistically significant. Analyses were performed with SPSS 18.0 Software (SPSS Inc., Chicago, IL, USA).

## Results

The 202 participants had a mean age of 30 years and 34% were females (Table 1). All patients met the ICD-10 criteria for SUD; the majority had a drug use disorder (83%). For 56% of participants, the mean GSI score (based on SCL-90) was above the cutoff value for psychiatric pathology.

### QoL at baseline

**Figure 1:**
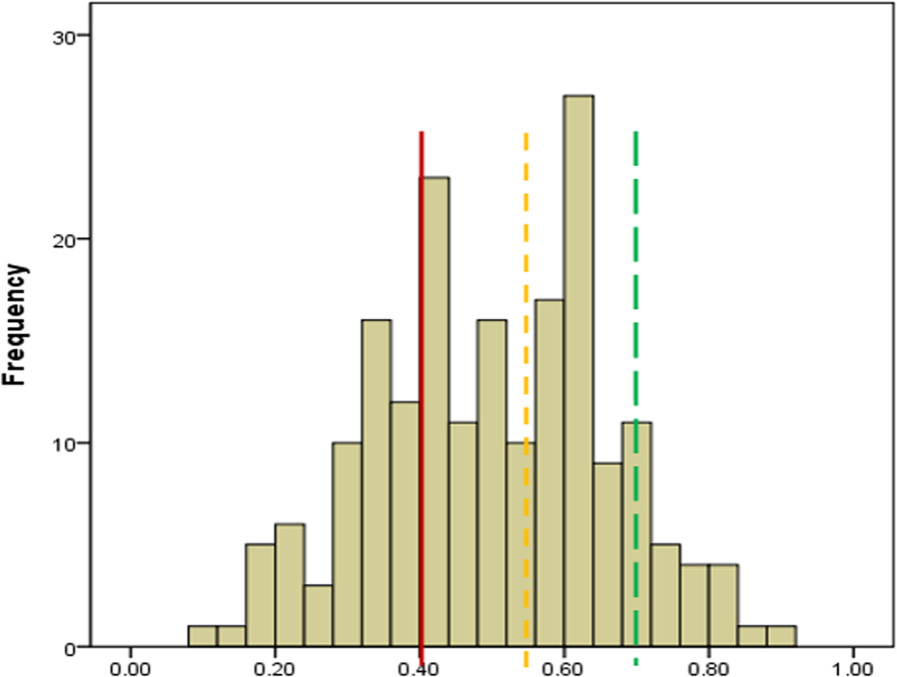
**Distribution of Quality of Life scores (QoL-5) at baseline (N=195).** The mean QoL-5 was 0.69 in normative data from a general population sample (green line); a value < 0.55 (yellow line) is considered a markedly reduced QoL; a value < 0.4 (red line) is considered a severely reduced QoL.

The QoL at baseline was low for a majority of patients; 59% had a QoL-5 score below 0.55 and 34% had an extremely low QoL (<0.40, Figure 1). In a linear regression model, psychiatric symptom distress (SCL90 - GSI) was the only factor significantly associated with QoL (Table 2). The SCL90 - GSI explained 39% of the variance in QoL. Thus, a high psychiatric symptom burden was associated with a low QoL at baseline. We did not find a significant association between substance use severity indices and Qol, and there were no evidence for a difference in QoL scores between the CA and VA groups (Table 2).

**Table 2:** **Linear regression analysis shows the effect of independent variables on quality of life at baseline in patients with substance use disorder (SUD), N=195 patients** ^**a**^

**Variable**	**B (95% CI)**	**P** ^**b**^	**R2** ^**c**^
Age	−0.01 (0.00/0.00)	0.553	
Female gender	−0.03 (−0.08/0.02)	0.268	
Education (years)	−0.01 (−0.02/0.05)	0.238	
Relationship status, single	0.01 (−0.04/0.06)	0.823	
Main SUD diagnosis	−0.02 (−0.08/0.04)	0.532	
Severity Scores			
Injection use	0.11 (−0.38/0.06)	0.671	
Years of using most problematic substance	0.00 (0.00/0.00)	0.942	
Global Score Index: SCL-90R - GSI	−0.15 (−0.18/-0.12)	<0.001	39%
Compulsory hospitalization	0.02 (−0.03/0.07)	0.413	

^a^Seven patients had missing QoL scores at intake.

^b^P-value obtained from bivariate linear regression. Only one independent variable had P-value <0.20 in bivariate analyses.

^c^R^2^= squared correlation coefficient in order to obtain a measure of explained variance.

### QoL at follow up 6 months after discharge from hospital

After 6 months, 58% of patients showed a positive change in QoL score. Improvements in QoL were classified as large (≥0.20) in 31 patients (26%); moderate (0.10 – 0.19) in 23 patients (19%); small in 8 patients (7%); and very small in 7 patients (6%). Forty-nine patients (42%) showed either no change or deterioration in QoL. The mean QoL-5 score change showed a significant, though modest, positive improvement of 0.06 (95% CI = 0.03 – 0.09, t=3.8, p<0.001, paired samples t-test) for the group as a whole. When analyzed separately the CA group showed a 0.05 mean QoL score improvement (95 % CI = 0.00 – 0.10, p = 0.055), which was of similar magnitude to that observed in the VA group (0.07, 95% CI = 0.03 – 0.11, p=0.001).

A logistic regression analysis was performed with a large QoL change (≥0.2) as the dependent variable (Table 3). The data offered no evidence for a difference between the CA and VA group in the bivariate analysis, the CA group had an OR 1.28 (95% CI = 0.56 – 2.94). The multivariate analysis only retained gender (females) as a predictor, with an OR of 2.64 (95% CI = 1.12 - 6.22, p=0.026).

**Table 3:** **Predictors of Quality of Life change** ^**a**^ **from baseline to follow-up, N=118 patients**

**Parameter**	**Bivariate analysis OR (95% CI)**	**P-value** ^**b**^	**Multivariate analysis OR (95% CI)**	**P-value** ^**c**^
Age	1.00 (0.96 – 1.05)	0.862	–	
Female gender	2.92 (1.12 – 6.71)	0.013	2.64 (1.12 - 6.22)	0.026
Education (years)	1.12 (0.92 –1.37)	0.269	–	
Relationship status, single	1.14 (0.43 – 2.80)	0.778	–	
Main diagnosis	0.93 (0.33 – 2.60)	0.891	–	
Severity scores				
Injection use	0.70 (0.31 – 1.53)	0.344	–	
Years of using most problematic substance	1.0 (0.96 – 1.01)	0.737	–	
Global Score Index: SCL-90R - GSI	1.72 (0.97 – 3.04)	0.064	1.52 (0.85-2.70)	0.157
Treatment variables				
Days in treatment	1.01 (0.10 – 1.02)	0.334	–	
Compulsory treatment	1.28 (0.56 – 2,94)	0.554		
Follow-up variables				
Abstinence at follow-up	1.51 (0.63 – 3.66)	0.356	–	
Time in a controlled environment (days)^d^	1.03 (0.98 1.03)	0.86	–	

^**a**^The dependent variable was a dichotomized QoL-5 score change, with a cut-off value of ≥ 0.2 (i.e., a large and clinically relevant QoL change).

^b^P-value obtained from bivariate logistic regression.

^c^P-value obtained from multivariable logistic regression; multivariable analysis included variables with p-values <0.20 in bivariate analyses.

^d^Time in controlled environment last 30 days before follow-up interview.

## Discussion

The majority of patients with SUD that were hospitalized had a seriously impaired QoL. A low QoL at baseline was associated with a high psychiatric symptom burden. At follow-up, the mean QoL score change showed a significant, though modest, positive improvement. Patients admitted either voluntarily or compulsorily had QoL improvements of similar magnitude. Female gender was associated with a clinically relevant improvement in QoL at follow-up.

In this study, we measured QoL with a generic instrument, the QoL-5, in hospitalized patients with SUD. The results showed a seriously impaired QoL at baseline. This finding corroborates previous available evidence, which showed that the QoL was consistently low among individuals with SUD that were actively seeking treatment, compared to the general population or individuals with other chronic health conditions [14]. Patients with SUD have observed QoL scores as low as or lower than those of patients with other chronic diseases and significantly lower than those of patients awaiting cardiac surgery [11,30].

We found that the psychiatric symptom burden correlated with the perceived QoL. This was somewhat expected, because mental function scores on QoL-scales were previously shown to be remarkably low for patients with SUD that were entering treatment, and on average, they were comparable to those found for patients with clinically-diagnosed depression [30]. Additionally, most patients with SUD that sought help also exhibited comorbid symptom disorders and/or personality disorders [31-33]. Somewhat unexpectedly, we found no association between the substance use severity indices and QoL. In a previous, large meta-analysis, the severity of dependence was the most powerful predictor of a low QoL [14]. The most unexpected finding was that the QoL scores were not different between the VA and CA groups. The NMHCA presupposes that the most serious cases would be those typically selected for compulsory treatment. Our findings implied that the selection of patients for compulsory hospitalization may not depend entirely on the severity factors; thus, other variables might be at play in the selection of these patients (e.g., an intervention by relatives that can put pressure on the social services to act) [34].

A literature search on compulsory hospitalization, though studies were quite limited, showed a tendency for improvement in the QoL following SUD treatment [35-37]. Our findings showed no evidence for a difference in QoL improvements between the CA and VA groups at the six-month follow-up. This similarity may be explained by the practice that the treatment for most patients in the CA group was integrated with that of the VA group as soon as possible. Other countries, like Sweden, use special institutions for patients hospitalized by CA, and they are not integrated with patients hospitalized by VA [38]. The quality of treatment is a crucial factor. Structured, integrated, and long-term treatment provide superior benefit to a “holding” strategy [38].

Compulsory hospitalization of patients with SUD is a controversial practice, both ethically and therapeutically. Many therapists in Norway point out that coercion reduces the patient’s control, freedom, and self-determination, and it threatens their autonomy. Therefore, the practice of CA requires strict regulations and documentation of positive outcomes. From an utilitarian perspective, it is necessary to weigh the pros and cons (i.e., the benefits and emotional costs for the patient) associated with this coercion [39]. Coercion should only be used when the pros outweigh, to some extent, the cons. This study has provided some preliminary evidence pointing towards beneficial outcomes also for compulsory treatment, which would be useful in an ethical debate with an utilitarian perspective.

At follow-up, we found that females showed larger improvements in QoL than males. All three wards included in the present study conducted a gender-mixed treatment program. Currently, in Norway, approximately 70% of patients in SUD treatment are men. Recently, woman-specific treatment has been advocated to improve outcomes for women [40,41]. Our findings indicated that the mixed-gender treatment provided greater improvements in QoL for women than for men.

In a review, Gerdner and Berglund point out that American studies show better outcome for CA than VA patients owing to better retention in treatment [38]. Swedish studies found no difference between these two groups. Similarly, we did not find a correlation between days in treatment and QoL outcome in our study.

Given the low QoL among patients with SUD that seek treatment, one would intuitively expect an association between reduced SUD symptoms and QoL improvement, and conversely that QoL would deteriorate among patients that relapsed [11]. Thus, it was unexpected in the present study to find that abstinence at follow-up was not a predictor for large improvements in QoL. However, the literature have reported mixed findings; some studies provided evidence that QoL improved with abstinence [42,43], but others found that there is not necessarily a link between the two [43,44]. For example, there was no correlation between a reduction in substance use and general life satisfaction among dually-diagnosed patients three years after assertive community treatment [44]. Those findings implied that improved QoL may not rely upon abstinence alone. In addition to reduced substance use, one should also focus on a broad range of factors that may underlie patient evaluation of QoL; most notably, important areas of recovery, like employment, housing, and means of social support; e.g., via mutual aid groups [45].

### Methodological considerations

This study had some limitations that should be considered when interpreting the results. There was a high attrition rate. However, the attrition analysis showed that there were no differences between those who dropped out and those who were included at follow-up with one exception; a larger proportion of CA patients was reached at follow-up. The higher drop-out in the VA group was due to administrative/logistic reasons. Thus, we do not believe that this has biased or reduced the generalizability of our findings. The follow-up rate of the CA group was quite respectable because this group was prioritized and the sample size was considered large enough for the performed regression analyses [46]. As the sample size and thus, power was smaller in the sample at follow-up than at baseline, these findings should be interpreted with caution. Self-reported information obtained from a QoL questionnaire poses a challenge in assessing experiences of the disease in a patient that was hospitalized by CA. Patients hospitalized by VA may generally be expected to be more cooperative than those hospitalized by CA. However, in this study, patients hospitalized by CA were not approached until they had “settled” down and had remained for some weeks in the wards; thus, they were considered competent for consent in participating in the study. It is not ethical to randomize to voluntary treatment patients that are deemed in need for compulsory treatment. Conversely; patients that are not deemed in need for compulsory treatment should not be randomized to a CA group. Thus, there were no random allocations of the participants in this study.

The study strengths were that this was, to our knowledge, the first study in Norway to assess clinical outcomes in patients hospitalized by CA. This study also reported longitudinal data.

## Conclusion

We showed that specialized SUD treatment improved QoL for patients with SUD. Our results also showed that females benefited more than males from a gender-mixed treatment paradigm. Our findings pointed to the usefulness of QoL measurements as evidence of therapeutic benefit in the recovery process in the SUD field.

## References

[1] Schipper H, Clinch JJ, Spilker B (1996). Quality of Life Studies: Definitions and Conceptual Issues. Quality of Life and Pharmacoeconomics in Clinical Trials.

[2] Oliver J, Huxley P, Bridges K, Mohamad H (1996). Quality of Life and Mental Health Services.

[3] Wood-Dauphinee S (1999). Assessing quality of life in clinical research: from where have we come and where are we going?. J Clin Epidemiol.

[4] Muldoon M, Barger S, Flory JD (1998). What are quality of life measurements measuring?. BMJ.

[5] McLellan AT, Kushner H, Metzger D, Peters R, Smith I, Grissom G (1992). The fifth edition of the addiction severity index. J Subst Abuse Treat.

[6] Venner KL (2006). Course of recovery from alcoholism. Alcohol Clin Exp Res.

[7] McLellan A, McKay JR, Forman R, Cacciola J, Kemp J (2005). Reconsidering the evaluation of addiction treatment: From retrospective follow-up to concurrent recovery monitoring. Addiction.

[8] Bonomi AE, Patrick DL, Bushnell DM, Martin M (2000). Validation of the United States’ version of the World Health Organization Quality of Life (WHOQOL) instrument. J Clin Epidemiol.

[9] Van den Brink W, Haasen C (2006). Evidenced-based treatment of opioid-dependent patients. Can J Psychiatry.

[10] McNeese-Smith DK, Wickman M, Nyamathi A, Kehoe P, Earvolino-Ramirez M, Robertson S (2009). Gender and ethnicity group differences among substance abuse treatment clients insured under managed care. J Addict Nurs.

[11] Laudet AB, Becker JB, White WL (2009). Don’t wanna go through that madness no more: quality of life satisfaction as predictor of sustained remission from illicit drug misuse. Subst Use Misuse.

[12] Raisch DW, Campbell HM, Garnand DA, Jones MA, Sather MS, Naik R (2012). Health-related quality of life changes associated with buprenorphine treatment for opioid dependence. Qual Life Res.

[13] Laudet AB (2011). The case for considering quality of life in addiction research and clinical practice. Addict Sci Clin Pract.

[14] Donovan D, Mattson M, Cisler R, Longabaugh R, Zweben A (2005). Quality of life as an outcome measure in alcoholism treatment research. J Stud Alcohol Suppl.

[15] Gonzales R, Ang A, Marinelli-Casey P, Glik DC, Iguchi MY, Rawson RA (2009). Health-related quality of life trajectories of methamphetamine-dependent individuals as a function of treatment completion and continued care over a 1-year period. J Subst Abuse Treat.

[16] Ministry of Health and Care Services (MHCS) (2011). The Municipal Health Care Act.

[17] Lundeberg IR, Mjåland K, Søvig KH (2014). Tvang i rusfeltet - Regelverk, praksis og erfaringer med tvang.

[18] Opsal A, Kristensen O, Larsen TK, Syversen G, Rudshaug EBA, Gerdner A (2013). Factors associated with involuntary admissions among patients with substance use disorders and comorbidity: a cross-sectional study. BMC Health Serv Res.

[19] Sheehan DV, Lecrubier Y, Sheehan KH, Amorim P, Janavs J, Weiller E (1998). The Mini-International Neuropsychiatric Interview (M.I.N.I.): the development and validation of a structured diagnostic psychiatric interview for DSM-IV and ICD-10. J Clin Psychiatry.

[20] Kokkevi A, Hartgers, C. EuropASI. European adaptation of a multidimensional assessment instrument for drug and alcohol dependence. Eur Addict Res. 1995;1(4):208-210.

[21] Derogatis LR (1986). SCL-90-R: Administration, Scoring And Procedures Manual for the R (evised) version and other Instruments of the Psychopathology Rating Scale Series.

[22] Derogatis LR, Lipman RS, Covi L (1973). SCL-90: An outpatient psychiatric rating scale, preliminary report. Psychopharmacol Bull.

[23] Lindholt JS, Ventegodt S, Henneberg EW (2002). Development and validation of QoL5 for clinical databases. A short, global and generic questionnaire based on an integrated theory of the quality of life. Eur J Surg.

[24] Ventegodt S, Merrick J, Andersen NJ (2003). Quality of life theory I. The IQOL theory: an integrative theory of the global quality of life concept. TheScientificWorldJOURNAL.

[25] Ventegodt S, Merrick J, Andersen NJ (2003). Measurement of quality of life II. From the philosphy of life to science. TheScientificWorldJOURNAL.

[26] Lindholt JS (2010). Mean QoL5 scores for Danish population.

[27] Ventegodt S (2010). QoL-5 - cut off Scores for Markedly and Severely Reduced QOL.

[28] Ventegodt S, Merrick J, Andersen NJ (2003). Editorial: a new method for generic measuring of the global quality of life. ThescientificWorldJOURNAL.

[29] Altman DG (1991). Practical Statistics for Medical Research.

[30] Smith KW, Larson MJ (2003). Quality of life assessments by adult substance abusers receiving publicly funded treatment in Massachusetts. Am J Drug Alcohol Abuse.

[31] Kessler RC (2004). Impact of substance abuse on the diagnosis, course, and treatment of mood disorders: the epidemiology of dual diagnosis. Biol Psychiatry.

[32] Bakken K, Landheim AS, Vaglum P (2007). Axis I and II disorders as long-term predictors of mental distress: A six-year prospective follow-up of substance-dependent patients. BMC Psychiatry.

[33] Ellingstad TP, Sobell LC, Sobell MB, Planthara P (2002). Drug treatment outcome methodology 1993–1997: Strengths, weaknesses, and a comparison to the alcohol field. Addict Behav.

[34] Lundeberg IR, Mjåland K (2009). Grenser for tvang. Om sosialtjenestens rolle i bruk av tvang overfor rusmiddelmisbrukere [The limits of coercion. The role of the social services in use of coercion against substance addicts].

[35] Liu T, Li L, Zhao M, Hao W (1998). A comparative study of the quality of life in heroin dependent patients. ChinJ Clin Psychol.

[36] Pollard JM (2007). Effects of differing types of perceived coercion on participants in jail diversion programs. Diss Abstr Int B Sci Eng.

[37] Freeman K (2003). Health and well-being outcomes for drug-dependent offenders on the NSW Drug Court programme. Drug Alcohol Rev.

[38] Gerdner A, Berglund M (2009). Compulsory Care for Substance Misuse – Effect and Quality; in Misuse, Knowledge and Care-Research Appendix of the Government Task Force on Substance Misuse SOU 2011:6.

[39] Torbjorn T (2002). Coercive Care: Ethics of Choice in Health & Medicine.

[40] Kringlen E, Torgersen S, Cramer V (2001). A Norwegian psychiatric epidemiological study. Am J Psychiatry.

[41] Duckert F, Lossius K, Ravdnal E, Sandvik B (2008). Kvinner og Alkohol ( Women and alcohol).

[42] Kraemer KL, Maisto SA, Conigliaro J, McNeil M, Gordon AJ, Kelley ME (2002). Decreased alcohol consumption in outpatient drinkers is associated with improved quality of life and fewer alcohol-related consequences. J Gen Intern Med.

[43] Villeneuve PJ, Challacombe L, Strike CJ, Myers T, Fischer B, Shore R (2006). Change in health-related quality of life of opiate users in low-threshold methadone programs. J Subst Use.

[44] McHugo GJ, Drake RE, Whitley R, Bond GR, Campbell K, Rapp CA (2007). Fidelity outcomes in the national Implementing Evidence-based Practices project. Psychiatr Serv.

[45] Vederhus JK, Timko C, Kristensen O, Hjemdahl B, Clausen T (2014). Motivational intervention to enhance post-detoxification 12-Step group affiliation: A randomized controlled trial. Addiction.

[46] Tabachnick BG, Fidell LS (2001). Using Multivariate Statistics.

